# Prevalence of household food insecurity in East Africa: Linking food access with climate vulnerability

**DOI:** 10.1016/j.crm.2021.100333

**Published:** 2021

**Authors:** Girma Gezimu Gebre, Dil Bahadur Rahut

**Affiliations:** a Faculty of Environment, Gender and Development Studies, Hawassa University, Hawassa, Ethiopia; b Department of Agricultural and Resource Economics, Graduate School of Bioresource and Bioenvironmental Sciences, Kyushu University, Fukuoka, Japan; c Asian Development Bank Institute, Tokyo, Japan; d International Maize and Wheat Improvement Center, El Batán, Texcoco, Mexico

**Keywords:** Food insecurity, Household, Climate change, Vulnerability, East Africa

## Abstract

The prevalence of food insecurity is much higher in East Africa than in other parts of the world. Climate change and associated variability are important contributors to food insecurity in the region. Using primary data collected in 2018/19 from Ethiopia, Kenya and Tanzania, this study examines the links between the prevalence of household food insecurity (the access to food dimension) and vulnerability to climate change in East Africa. The Household Food Insecurity Access Scale (HFIAS) was constructed to measure the prevalence of household food insecurity, and an ordered probit econometrics model was used to investigate the factors affecting the prevalence rates. The aggregate results show that 52% of the total sampled households in the region were food-secure; 15% and 26% were mildly food-secure and moderately food-insecure, respectively; and the remaining 7% were severely food-insecure. The ordered probit results suggest that exposure to climate change extremes and crop losses caused by these extremes significantly contribute to the prevalence of food insecurity across countries in East Africa. The results also indicate that households’ adaptive capacity plays a significant role in reducing the prevalence of food insecurity. The demographic/human, social, financial, physical, and natural assets/capital of the household also play a significant role in reducing household-level food insecurity in Ethiopia, Kenya, and Tanzania.

## 1 Introduction

The 2030 Agenda for Sustainable Development, adopted by the United Nations General Assembly on 25 September 2015, aims to “end hunger and ensure access by all people, in particular, the poor and people in vulnerable situations, including infants, to safe, nutritious and sufficient food all year round” (Food and Agriculture Organization, (FAO), 2017). However, food security remains a major uncertainty for households and individuals across the globe (Agidew and Singh, 2018, Abdullah et al., 2019, Drammeh et al., 2019, Kabubo-Mariara and Mulwa, 2019, Thome et al., 2019, Oduniyi and Tekana, 2020). The recent estimates by FAO et al. (2019) indicate that 26.4% of the world population were living in a food-insecure state in 2018.

The prevalence of food insecurity is much higher in Africa than in other parts of the world (FAO, 2017, Smith et al., 2017, Drammeh et al., 2019, Kabubo-Mariara and Mulwa, 2019). More than 50% of the African population is exposed to moderate or severe food insecurity (FAO, Ifad, UNICEF, WFP, & WHO, 2019, Thome et al., 2019). In 2018, the highest level of food insecurity was recorded in East Africa (63% of the population, or 272 million), followed by southern Africa (54% or 35 million), West Africa (48% or 183 million), and North Africa (30% or 70 million) (FAO et al., 2019). This implies that most of the East African population does not have regular access to nutritious and sufficient food for a healthy and productive life. Three main reasons have been identified by the FAO (2017) for the rise of food insecurity in East Africa: violent conflicts, climate adversity, and the global economic environment. Using rainfall data collected between 1960 and 2016 from 71 developing countries, Kinda et al. (2019) concluded that the negative effects of climate change such as rainfall variability are exacerbated in the presence of conflicts, and are high for the countries that are vulnerable to food price shocks. A report by FAO et al. (2018), which focused exclusively on climate change and food security, noted that severe food insecurity is significantly worse in countries with agricultural systems that are highly sensitive to rainfall and temperature variability and severe drought, and where the livelihood of a high proportion of the population depends on agriculture.

The negative effects of climate change and associated variability in East Africa are more severe due mainly to the interaction of multiple factors, including high population growth, extreme poverty, poor infrastructure, overdependence on rain-fed agriculture, poor availability and quality of meteorological data, and knowledge gaps (IPCC, 2007, Agidew and Singh, 2018, Kabubo-Mariara and Mulwa, 2019, Drammeh et al., 2019). These factors contribute to weak adaptive capacities of the countries in the region (Kabubo-Mariara and Mulwa, 2019). In addition, socio-economic factors such as low levels of education, weak social networks, limited social capital, unemployment and low household income are the major contributors to food insecurity in the region (Smith et al., 2017) and are thus sufficient to render farm households incapable of adapting to climate change (Drammeh et al., 2019). Hence, our focus in this paper is to examine the prevalence of household food insecurity (access to food) in the face of vulnerability to climate change in East Africa. This is an area where significant knowledge gaps prevail, and the 2018 UN world food security report (FAO et al., 2018) has called for further research. In line with this, our study answers the following questions: (1) What is the prevalence of food insecurity among rural households in Ethiopia, Kenya, and Tanzania? (2) How does vulnerability to climate change affect the prevalence of different levels of household food insecurity in Ethiopia, Kenya, and Tanzania?

Climate change predictions for sub-Saharan Africa suggest reduced rainfall, increased erratic rainfall, intra-seasonal dry spells and incidences of flooding, high temperatures, and higher frequency of droughts (Hadebe et al., 2016). Climate variability has increased the frequency and intensity of extreme events such as droughts and floods across countries in East Africa (Nicholson, 2017, Mbow et al., 2019), and people who are already vulnerable and food insecure are likely to be the first affected by these (FAO et al., 2020). Such extremes worsen the food security situation of smallholders and subsistence farm households in the region who have difficulty in providing enough food for their members (Shisanya and Mafongoya, 2016, Schanzenbach et al., 2016). In this context, studying food insecurity in the face of climatic adversity may help us understand the underlying factors that contribute to the prevalence of food insecurity (limited access to food) in East Africa, which is the most fragile and drought-prone area in the world.

## 2 Conceptual framework and measurement

### 2.1 Conceptual framework

Food security is defined by the FAO as “a situation that exists when all people, at all times, have physical, social, and economic access to sufficient, safe and nutritious food that meets their dietary needs and food preferences for active and healthy life” (FAO, 1996). In contrast, food insecurity is defined by the FAO as “a situation that exists when people lack secure access to sufficient amounts of safe and nutritious food for normal growth and development, and active and healthy life.” (ibid). As the FAO definition of food insecurity places emphasis on access to food, our study links the food access dimension of food insecurity with vulnerability to climate change. Food access is a function of the demographic, social, physical, and policy environments that determine how effectively households are able to utilize their resources to meet their food needs (Shah and Dulal, 2015, Sam et al., 2019). These demographic, social, physical, and policy settings that determine food access change with climate vulnerability (Schmidhuber and Tubiello, 2007, Sam et al., 2019).

The term “vulnerability” has no universally accepted definition (Cutter et al., 2009, IPCC, 2007, Nelson et al., 2010). Hence, this study refers to the widely-cited definition provided by the Intergovernmental Panel on Climate Change (IPCC), which defines vulnerability as “the degree to which an environmental or social system is susceptible to, and unable to cope with, adverse effects of climate change, including climate variability and extremes” (IPCC, 2007). Vulnerability is a result of a system’s exposure and sensitivity to climatic stimuli, and its capacity to adapt to their adverse effects (IPCC, 2007). As defined by the IPCC, exposure refers to the presence of people with their livelihoods, resources, infrastructure, and economic, social, and cultural assets, in places that could be adversely affected by climate change outcomes such as extremes of rainfall (drought or flood). Sensitivity refers to the degree to which a system is negatively affected by climate variability or drought. Adaptive capacity refers to a system’s ability to respond successfully to the adverse effects of climate change. When these functions are described at the household level, food (in)security falls under the sensitivity function (Sam et al., 2019); however, this paper argues that the prevalence of agrarian household food insecurity (the food access dimension) could be determined by the loss of or decline in household food production due to damage from climate change extremes such as drought or flooding. That is, both loss or decline in household crop production and household food insecurity would be considered under the sensitivity dimensions of climate change vulnerability, since loss or decline in crop production leads to insufficient access to food in the household.

**Fig. 1:**
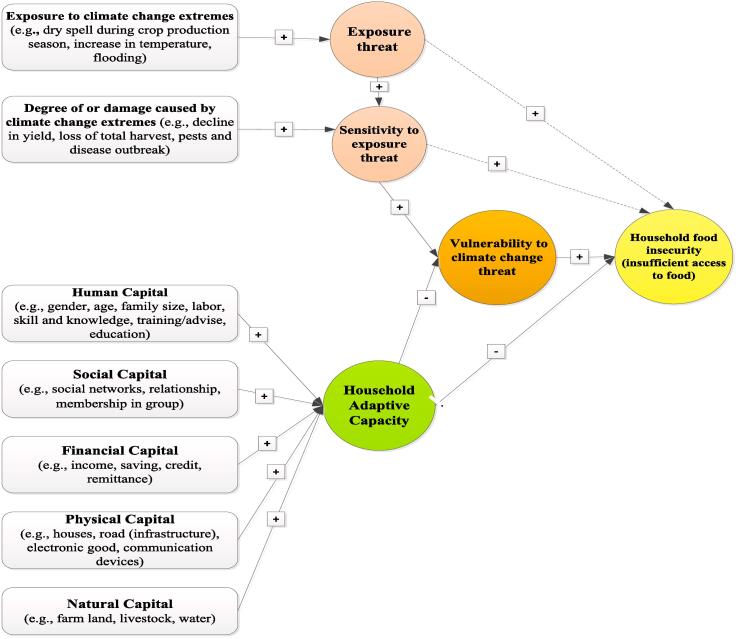
Conceptual framework of household food insecurity (food access) in the context of the IPCC dimensions of vulnerability (i.e., the links between climate exposure threat, sensitivity, adaptive capacity, and lack of access to sufficient food for a household). Note that the arrows and the ± signs indicate expected direction of effects on the corresponding variables or indicators; the dashed line indicates a potential impact (excluding adaptive capacity).

The adaptive capacity or ability of households to respond to their food-insecure state depends on their human, social, financial, natural, and physical endowments (Antwi-Agyei et al., 2013, Wright et al., 2012, Islam and Al Mamun, 2020, Piya et al., 2016, Sam et al., 2019). A household with greater adaptive capacity is less likely to be rendered food insecure. In considering the above situations and following Islam and Al Mamun (2020), this study conceptualizes the links between food insecurity (food access) and climate change vulnerability (exposure, sensitivity, and adaptive capacity) as in Fig. 1.

### 2.2 Measurements of Household-level food Security/Insecurity

As food security is a complex and multidimensional concept that encompasses availability, access, utilization and stability, a single measure cannot capture all the dimensions of what it means to be food secure or food insecure (see, e.g., Coates and Maxwell, 2012, Coates, 2013, Jones et al., 2013, Tadesse et al., 2020). Therefore, analysts use a diverse set of proxy indicators that capture different dimensions of food security/insecurity. These are broadly divided into objective and subjective/experimental measures. The objective measures include calorie intake or availability, monetary poverty thresholds, and dietary diversity (Headey and Ecker, 2012, Tadesse et al., 2020). These measures are based on a consumption or income approach. Consumption data tend to have large seasonal volatility, and such data are most often collected through a single-round survey undertaken in a specific month of the year. Household surveys of food (in)security studies based on consumption data usually focus on either the four-week or seven-day duration prior to the start of survey interviews. Hence, consumption data could be subjected to problems related to infrequent purchase (where, for example, food is purchased shortly after the survey interview, consequently biasing the results of reported expenditure in the month prior to the interview). This could be coupled by measurement errors related to respondents‘ imperfect recall or reporting bias. Accordingly, consumption data may systematically under-or over-report the actual food (in)security situation, depending on the time of the year the survey is conducted (Gebre et al., 2021). Therefore, the objective indicators have limited use for evaluating the seasonality of food insecurity, including the impact of shocks, unless one resorts to an expensive solution such as collecting high-frequency data (Tadesse et al., 2020). Some datasets such as the Living Standards Measurement Study (LSMS) and Demographic Health Surveys (DHS) allow objective indicators of food security to look at changes across time, which then allows insight into seasonality.

The subjective or experimental measures of food insecurity rely on the respondent’s perception or experience of the availability and access to adequate food. Respondents are asked about the occurrence or frequency of occurrence of the food insecurity that they (individually or as a household) have experienced. Therefore, unlike objective indicators, subjective indicators are constructed based on self-reported responses to a series of direct questions related to the shortage of food and its consequences. They measure the severity as well as the full extent of food insecurity, from the psychological to the more physical feelings of the respondent (Headey and Ecker, 2012). Subjective food insecurity indicators include simple dichotomous indicators, such as the Food Insecurity Experience Scale (FIES) developed by the United Nations Food and Agriculture Organization’s Voice of the Hungry project. The FIES asks whether respondents have experienced a problem in affording food over the previous one-month or twelve-month period, depending on the research or programmatic priorities. The FIES consists of eight questions capturing a range of food-insecurity severity, with yes/no responses. These questions focus mainly on the occurrence of food insecurity in the recall period and not on the frequency of occurrence within the stated recall period.

Another sophisticated experimental indicator of food insecurity is the Household Food Insecurity Access Scale (HFIAS) that was developed by the United States Agency for International Development (USAID) Food and Nutrition Technical Assistant (FANTA) project. The HFIAS indicator is based on the assumption that the experience of food insecurity (access to food) causes predictable reactions and responses that can be captured and quantified through a survey and summarized on a scale (Coates et al., 2007, Headey and Ecker, 2012). In the HFIAS approach, respondents are asked nine questions related to the occurrence of food insecurity and the frequency of occurrence over a four-week recall period. The occurrence questions represent an increasing level of severity of food insecurity, and the frequency-of-occurrence questions are asked as a follow-up to each occurrence question to determine how often the condition occurred. For instance, if the respondent answers “yes” to an occurrence question, a frequency-of-occurrence question is asked to determine whether the condition happened rarely (once or twice), sometimes (three to ten times) or often (more than ten times) in the past four weeks (30 days) (Coates et al., 2007, Chinnakali et al., 2014). The HFIAS approach yields information on food insecurity (access to food) at the household level.

This paper uses the HFIAS to measure the prevalence of household food insecurity in East Africa. Specifically, the paper uses the Household Food Insecurity Access Prevalence (HFIAP) indicator to evaluate the prevalence levels of food insecurity. The HFIAP indicator categorizes households into four levels: food-secure, mildly food-insecure, moderately food-insecure, and severely food-insecure.

Following Coates et al. (2007), the operational definitions of household food insecurity used in this study are as follows: *Food-secure* describes a situation where a household experiences none of the food insecurity (access to food) conditions, or just experiences anxiety, but only rarely. *Mildly food-insecure* describes the situation where a household worries about not having enough food sometimes or often, and/or is unable to eat preferred foods, eats a more monotonous diet than desired, and/or consumes some foods considered undesirable, but only rarely; however, the household does not cut back on quantity, nor does it experience any of the three most severe conditions: running out of food, going to bed hungry, or going a whole day and night without eating. *Moderately food-insecure* describes a situation where a household sacrifices quality more frequently, by eating a monotonous diet or undesirable foods sometimes or often, and/or has started to cut back on quantity by reducing the size of meals or the number of meals, rarely or occasionally. *Severely food-insecure* describes a situation where a household has graduated to cutting back on meal size or the number of meals often, and/or experiences any of the three most severe conditions (running out of food, going to bed hungry, or going a whole day and night without eating), even as infrequently as rarely.

## 3 Study area, Data, and methods of analysis

### 3.1 Study area

The East Africa region is the most vulnerable in the world to climate-related risks. Apart from protracted conflict and political violence, climate-induced risk is the major driver of vulnerability in the region, particularly for poor communities whose livelihood depends on rainfed agricultural systems (FAO, 2017), which account for 40% of the regional economy. Extreme weather events that are associated with climate change and variability have increased in recent years. This has resulted in more crop and livestock diseases, livestock deaths, and total crop losses, as well as frequent emergencies, food insecurity, infrastructure damage, and high economic costs. On the other hand, East Africa is a region with one of the highest population growths in the world (Waithaka et al., 2013), and as a result, the demand for food is increasing dramatically. This study focuses on Ethiopia, Kenya, and Tanzania that together make up a significant share of the region’s population, while experiencing frequent droughts related to climate change.

### 3.2 Data

The study used the primary data that was collected in 2018/19 from Ethiopia, Kenya, and Tanzania using a purposive multi-stage sampling strategy. In Ethiopia, the data were collected from the three biggest regional states in the country: Amhara, Oromia and the South Nation, Nationalities, and People (SNNP) regional states. In Amhara region, data were collected from Awi and West Gojam zones; in Oromia region, data were taken in Jimma, East Shewa, Aris, and West Aris zones; and in the SNNP region, data were collected from Gurage, Hadiya, Wolaita and, Gamo zones. In Kenya, the data were recorded in Makueni, Machakos, Embu, Tharaka Nithi, Kakamega, and Busia counties, while in Tanzania, data were collected from Morogoro, Iringa, Mbeya, Tabora, Manyara, Simiyu, Arusha, and Kilimanjaro regions. In total, data were collected from 1912 smallholder farm households (516 in Ethiopia, 676 in Kenya, and 720 in Tanzania). The collected data had detailed information on household demographic characteristics, social networks, households’ assets, climate change and adaptation to it by the household, food security, location, incomes, agricultural output, and other institutional information.

### 3.3 Methods of analysis

Prevalence levels of household food insecurity were calculated using the HFIAP indicators. Firstly, we coded frequency-of-occurrence as 0 for all cases where the answer to the corresponding occurrence question was “no” (i.e., if the answer to Q_1_ was “no” then frequency-of-occurrence was coded as Q_1_ = 0 and so on). If the answer to the occurrence question was “yes”, then a frequency-of-occurrence question was coded as 1 for all cases where the situation occurred rarely, 2 for sometimes, and 3 for often. In short, each occurrence question (Table 1) was assigned four alternative codes (e.g., Q_1_ was coded as Q_1_ = 0 for no occurrence, Q_1_ = 1 for rare occurrence, Q_1_ = 2 for occasional occurrence_,_ or Q_1_ = 3 for frequent occurrence). Secondly, the Household Food Insecurity Access (HFIA) category variable was calculated for each household using the assigned codes of the degree of food insecurity in which it fell. Accordingly, four categories of food insecurity were created sequentially, (1 = food secure, 2 = mildly food insecure, 3 = moderately food insecure, and 4 = severely food insecure), to ensure that households were classified according to their most severe response.

**Table 1:** Questions in the Household Food Insecurity Access Prevalence.

No.	Occurrence Questions
Q_1_	In the past four weeks, did you worry that your household would not have enough food?
Q_2_	In the past four weeks, were you or any household member not able to eat the kinds of foods you preferred because of a lack of resources?
Q_3_	In the past four weeks, did you or any household member have to eat a limited variety of foods due to a lack of resources?
Q_4_	In the past four weeks, did you or any household member have to eat some foods that you really did not want to eat because of a lack of resources to obtain other types of food?
Q_5_	In the past four weeks, did you or any household member have to eat a smaller meal than you felt you needed because there was not enough food?
Q_6_	In the past four weeks, did you or any household member have to eat fewer meals in a day because there was not enough food?
Q_7_	In the past four weeks, was there ever no food to eat of any kind in your household because of a lack of resources to get food?
Q_8_	In the past four weeks, did you or any household member go to sleep at night hungry because there was not enough food?
Q_9_	In the past four weeks, did you or any household member go a whole day and night without eating anything because there was not enough food?

Following the operational definition of food insecurity used in this paper, each category of the HFIA was calculated from Table 1 as:

$$\mathrm{Food}\;\sec ure\;\;=1\;if\left[\left(Q_{1}=0or\;Q_{1}=1\right)and\;Q_{2}=0\;and\;Q_{3}=0\;and\;Q_{4}=0and\;Q_{5}=0\;and\;Q_{6}=0and\;Q_{7}=0and\;Q_{8}=0\;and\;Q_{9}=0\right]$$

$$\begin{array}{c} \;Mildly\;food\;in\sec ure=2\;if\;[\left(Q_{1}=2\;or\;Q_{1}=3\;or\;Q_{2}=1\;or\;Q_{2}=2\;or\;Q_{2}=3\;or\;Q_{3}=1\;or\;Q_{4}=1\right)and\;Q_{5}\\ =0\;and\;Q_{6}=0\;and\;\;\;Q_{7}=0\;and\;Q_{8}=0\;and\;Q_{9}=0] \end{array}$$

$$\mathrm{Moderately}\;food\;in\sec ure=3\;if\;\left[\left(Q_{3}=2\;or\;Q_{3}=3\;or\;Q_{4}=2\;or\;Q_{4}=3\;or\;Q_{5}=1\;or\;Q_{5}=2\;or\;Q_{6}=1\;or\;Q_{6}=2\right)and\;Q_{7}=0\;and\;Q_{8}=0\;and\;Q_{9}=0\right]$$

Severelyfoodinsecure=4

if [Q_5_ = 3 or Q_6_ = 3 or Q_7_ = 1 or Q_8_ = 2 or Q_7_ = 3 or Q_8_ = 1 or Q_8_ = 2 or Q_9_ = 3 or Q_9_ = 1 orQ = 2 or Q_9_ = 3] (1)

Finally, the prevalence of the different levels of household food insecurity was calculated by dividing the number of households in one category to the total number of households in the four categories.

To examine the effect of climate vulnerability on the prevalence of the different levels of household food insecurity, an ordered probit model was estimated to derive the probabilities related to the prevalence of household food insecurity. This was because the outcome variables (food secure, mildly food-insecure, moderately food-insecure, and severely food-insecure) were categorical and ordinal.

Following Greene (2012), the standard ordered probit equation is given as:

(2) $$Y_{i}^{*}=X_{i}\beta +\varepsilon_{i},\varepsilon_{i}|X_{i}\sim Normal\left(0,\;1\right)$$

where, $Y_{i}^{*}$ is a latent variable, i represents food-secure, mildly food-insecure, moderately food-insecure, or severely food-insecure farm households; $X_{i}$ is a vector of explanatory variables describing climate change vulnerability (exposure, sensitivity, and adaptive capacity); β is the coefficient and $\varepsilon_{i}$ is the random error term assumed to be normal. Let $\alpha_{1},<\alpha_{2},<\alpha_{3}$, be the unknown threshold parameters (cut-off points), and define the probability of the household food (in)security status in Equation (2) as:

(3) $$Y_{i}=\left\{\begin{array}{c} 1\;\;if\;\;\;\;\;Y_{i}^{*}\;⩽\alpha_{1},\;food\;\sec ure\\ 2\;\;if\;\;\;\;\;\;\alpha_{1}<Y_{i}^{*}\;⩽\alpha_{2}\;\;\;\;\;\;\;\;\;\;\;\;\;\;mildly\;food\;in\sec ure\\ 3\;\;if\;\;\;\;\;\alpha_{2}\;<Y_{i}^{*}\;⩽\alpha_{3}\;\;\;\;\mathrm{mod}\;erately\;food\;in\sec ure\\ 4\;\;if\;\;\;\;\;\;\;Y_{i}^{*}\;>\alpha_{3}\;severely\;food\;in\sec ure \end{array}\right)$$

As observed by Greene (2012), the coefficient (β) estimates from the ordered probit regression are not straightforward and are difficult to interpret. The coefficient estimates simply give the direction of the explanatory variables on the outcome variables. They do not represent the actual magnitude of change associated with the explanatory variables. Thus, the marginal effects of each explanatory variable on the probabilities are discussed in this paper. The description of the variables used in the model analysis is provided in table 2.

**Table 2:** Description of variables used in the ordered probit regression.

Variables	Description and measurement	Expected sign
Dependent variable		
Food insecurity	1 = Food-insecure	
	2 = Mildly food-insecure	
	3 = Moderately food-insecure	
	4 = severely food-insecure	
Independent Variables		
Exposure		
Latest period dry spell	Frequency of dry spells that impacted major crop production between 2016 and 2018.	+
Longer period dry spell	Frequency of dry spells that impacted major crop production over the last ten years (2008–2018)	+
Sensitivity		
Crop loss	Number of seasons the household lost their crop due to dry spells between 2016 and 2018.	+
Household Adaptive Capacity		
Human Capital		
Gender	Sex of the household head (1 = male)	–
Age	Age of the household head in years	-/+
Family size	Number of household’s members	-/+
Education level	Education level of the head in years	–
Reciprocal labor	Does household use/access reciprocal labor (1 = yes)	–
Financial Capital		
Saving	Does current income allow household to save money (1 = yes)	–
Borrowings	Is the household able to borrow money from formal and informal sources (1 = yes)	–
Social Capital		
Visiting village demonstration	Does household attend farm demonstration sites in respondent’s village (1 = yes)	–
Visiting another village	Does household attend farm demonstration sites in another village (1 = yes)	–
Extension contact	Number of contacts with extension agent in 2018.	–
Information on rainfall and temperature	Does household regularly receive information on expected rainfall and temperature (1 = yes)	–
Membership in social group	Is household a member of a social group such as a farmers’ cooperative or farmers’ association? (1 = yes)	–
Physical Capital		
Distance to Market	Distance to the nearest main crop market in KM?	–
Distance to agriculture office	Distance to nearest government agricultural field office in KM?	–
Owned radio	Does the household currently own any radio or television? (1 = yes)	–
Natural Capital		
Land size	Total farm size owned by the household in hectares	–
Livestock	Number of livestock owned by the household in tropical livestock units (TLU)	–

Note that the expected sign was predicted from the perspective of food insecurity.

## 4 Results and discussion

### 4.1 Descriptive results

Table 3 provides the descriptive statistics of the prevalence rates of household food insecurity. Of the total surveyed farm households in the three countries, 52% were food-secure, while 15% and 26% were mildly food-insecure and moderately food-insecure, respectively; the remaining 7% of households were severely food-insecure. These results indicate that about 52% and 48% of the total sampled households in East Africa are food-secure and food-insecure, respectively. The prevalence rates of the moderately and severely food-insecure states, in our findings, are lower than the findings reported by FAO et al. (2020) in the East African region. They found that about 26% of people in East Africa were severely food-insecure in 2018, while 37% of people in the region were moderately food-insecure in the same year. The FAO thus concluded that about 63% of East Africans did not have regular access to nutritious and sufficient food, even if they were not necessarily suffering from hunger. The FAO analysis did not consider mildly food-insecure households; this might thus be a reason for the lower prevalence of moderately food-insecure and severely food-insecure households in this study.

**Table 3:** Prevalence of household food insecurity by country.

Household food insecurity level	Aggregate (N = 1,912)	Ethiopia (N = 516)	Kenya (N = 676)	Tanzania (N = 720)
Food-secure	52%	60%	50%	48%
Mildly food-insecure	15%	11%	12%	22%
Moderately food-insecure	26%	22%	30%	24%
Severely food-insecure	7%	7%	8%	6%

Country-specific analysis shows that about 60% of the surveyed farm households in Ethiopia were food-secure, while 11% and 22% of the households were mildly food-insecure and moderately food-insecure, respectively. Of the Kenyan households, 50% were food-secure, while 12% and 30% were mildly food-insecure and moderately food-insecure, respectively. In Tanzania, 48% of the farm households were food-secure, while 22% and 24% were mildly food-insecure and moderately food-insecure, respectively. These results indicate that there is a slight variation in the prevalence rates of farm household food insecurity across countries in the East African region.

Table 4 provides descriptive statistics of the explanatory variables used in the model analysis. Different explanatory variables were included to predict the influence of climate change vulnerability on the prevalence levels of household food insecurity. They mainly include variables related to exposure to climate change extremes such as short and long dry spells, degree of damage or loss of crop production due to dry spells, and a household’s ability to respond to damage from climate change-related extremes. Aggregate results show that all the food-insecure categories of the households suffered more adversity from climate change extremes; however, the severity of the damage due to these was higher in the moderately and severely food-insecure categories. The country-specific results show that Kenya recorded more frequent droughts in the last three years than Ethiopia and Tanzania; however, over a longer period, the last ten years, all three countries recorded nearly similar occurrences of dry spells during the major production seasons.

**Table 4:** Summary statistics showing the average values of the included variables by the prevalence of different levels of food insecurity.

Variables	Aggregate				Ethiopia				Kenya				Tanzania			
	Food-secure	Mild	Moderate	Severe	Food-secure	Mild	Moderate	Severe	Food-secure	Mild	Moderate	Severe	Food-secure	Mild	Moderate	Severe
Exposure																
Latest dry spell	0.84	0.97	1.17	1.38	0.94	0.91	0.88	1.18	0.97	1.41	1.37	1.88	0.61	0.96	1.16	1.25
Longer dry spell	1.11	1.57	1.77	1.78	1.35	1.55	1.83	1.97	1.43	1.68	1.71	1.85	1.38	1.75	1.76	1.98
Sensitivity																
Crop loss	0.70	0.88	0.96	0.98	0.64	0.87	0.94	0.95	0.57	1.40	1.11	1.08	0.68	0.99	0.83	1.28
Adaptive Capacity																
*Haman Capital*																
Gender (Male head)	0.85	0.84	0.80	0.72	0.94	0.91	0.90	0.80	0.75	0.69	0.64	0.57	0.95	0.88	0.72	0.64
Age	47.30	51.56	50.71	48.0	49.50	51.02	46.48	48.97	40.62	50.04	52.15	44.69	52.23	52.55	52.00	51.40
Family size	5.30	5.80	6.33	7.24	6.87	6.91	7.00	6.74	3.72	5.06	5.97	8.77	5.41	5.83	6.33	7.73
Education level	7.73	6.13	5.58	5.60	5.09	4.93	3.21	3.09	9.30	8.56	7.65	5.70	7.06	6.70	6.50	4.15
Reciprocal labor	0.61	0.50	0.72	1.02	1.91	1.63	1.33	1.27	0.14	0.12	0.43	1.35	0.44	0.35	0.19	0.23
*Financial Capital*																
Savings	0.45	0.24	0.20	0.20	0.62	0.26	0.19	0.12	0.19	0.08	0.11	0.10	0.56	0.40	0.31	0.20
Borrowings	0.36	0.35	0.30	0.20	0.31	0.40	0.18	0.15	0.33	0.41	0.38	0.20	0.43	0.30	0.28	0.25
*Social Capital*																
Visiting village demonstration site	0.38	0.29	0.34	0.30	0.85	0.77	0.74	0.61	0.19	0.15	0.18	0.20	0.27	0.21	0.20	0.05
Visit another village	0.14	0.12	0.14	0.14	0.20	0.18	0.24	0.18	0.12	0.07	0.14	0.16	0.11	0.12	0.06	0.08
Extension contact	1.11	0.92	0.84	1.43	2.42	2.93	2.80	2.94	0.32	0.20	0.23	0.14	0.71	0.63	0.23	1.80
Information	0.70	0.58	0.50	0.43	0.79	0.64	0.60	0.42	0.76	0.66	0.56	0.68	0.54	0.47	0.49	0.25
Membership in group	0.48	0.36	0.30	0.25	0.56	0.48	0.37	0.31	0.51	0.36	0.24	0.28	0.26	0.20	0.10	0.10
*Physical Capital*																
Distance to Market	6.31	6.32	6.48	6.10	8.02	9.50	7.56	8.03	2.77	2.80	3.14	2.24	8.23	7.14	9.67	9.40
Distance to Agri Office	4.92	3.86	3.63	3.91	3.30	2.72	2.72	2.63	6.19	4.99	4.63	4.87	3.38	3.11	4.42	3.77
Owned radio or TV	0.75	0.70	0.61	0.42	0.56	0.40	0.24	0.15	0.88	0.81	0.69	0.59	0.83	0.80	0.63	0.43
*Natural Capital*																
Land size (ha)	2.47	2.91	2.19	1.82	1.97	1.91	1.81	2.00	1.83	2.85	1.72	1.16	3.55	3.30	3.03	2.52
Livestock (TLU)	3.08	2.71	2.17	1.61	4.64	4.20	2.52	2.12	1.87	1.56	1.28	0.81	3.17	3.00	2.67	2.20
No. of observations	994	305	488	125	309	55	118	34	337	87	201	51	348	163	169	40

NB: TLU conversion factor cattle = 0.70, donkey = 0.50, goat and sheep = 0.10, and chicken = 0.01.

The survey results also show that in each country there was a higher proportion of male-headed households in the food-secure category and a lower proportion in the severely food-insecure category. This result implies that, without indicating any causal relationship, the prevalence levels of food insecurity are higher in female-headed households in East Africa. The results also show that in all three countries, the family size in food-secure households was small, while the family size in severely food-insecure households was larger. The average number of livestock owned, measured in TLU, was higher among food-secure households, while it was lower in severely food-insecure households in each country.

### 4.2 Factors affecting the prevalence of household food insecurity

The results of the factors affecting the prevalence of food insecurity among the surveyed farm households are presented in Table 5. We estimated aggregate and specific country samples to examine the effects of climate vulnerability on the prevalence status of household food insecurity. In both estimations, the model was significant at a 1% level, meaning that the explanatory variables taken together explain the prevalence status of household food insecurity. Since the coefficients from ordered probit regression do not tell us if there is a change in household food insecurity status due to a change in an explanatory variable, the marginal effects are discussed in this section. These marginal effects are interpreted based on the sign and category. The results offer insights into the linkage of vulnerability to climate change (the exposure, sensitivity, and adaptive capacity dimensions) to the prevalence of household food insecurity (access to food dimension).

**Table 5:** Average Marginal effects after ordered probit regression factors affecting the prevalence of household-level food insecurity.

Variables	Three countries combined (Aggregate)				Ethiopia				Kenya				Tanzania			
	Food-secure	Mild	Moderate	Severe	Food-secure	Mild	Moderate	Severe	Food-secure	Mild	Moderate	Severe	Food-secure	Mild	Moderate	Severe
Exposure																
Latest dry spell	−0.005*** (0.010)	0.002** (0.002)	0.003*** (0.005)	0.007*** (0.011)	−0.048 *** (0.035)	0.006** (0.004)	0.051 ** (0.021)	0.022*** (0.012)	−0.011*** (0.032)	0.001 (0.002)	0.007*** (0.021)	0.003*** (0.010)	0.019*** (0.010)	0.001* (0.002)	0.005** (0.006)	0.002*** (0.002)
**Longer dry spell**	−0.012*** (0.058)	0.001*** (0.001)	0.003*** (0.005)	0.003*** (0.002)	−0.007*** (0.015)	0.001*** (0.001)	0.004*** (0.009)	0.002*** (0.005)	−0.043*** (0.011)	0.002** (0.001)	0.027*** (0.007)	0.013*** (0.004)	−0.049*** (0.013)	0.008 *** (0.003)	0.029*** (0.008)	0.012 *** (0.003)
Sensitivity																
Crop loss	−0.011*** (0.015)	0.004** (0.002)	0.006*** (0.009)	0.003**** (0.004)	−0.140*** (0.045)	0.013*** (0.005)	0.082*** (0.027)	0.045** (0.016)	−0.042*** (0.029)	0.002* (0.002)	0.027** (0.018)	0.013*** (0.009)	−0.067*** (0.028)	0.011** (0.005)	0.040 ** (0.017)	0.016** (0.007)
Adaptive Capacity																
*Human Capital*																
Gender (Male head)^1^	0.010 (0.026)	−0.001 (0.003)	−0.006 (0.016)	−0.003 (0.007)	0.149** (0.062)	−0.014** (0.007)	−0.087** (0.036)	−0.048** (0.021)	0.001 (0.035)	−0.000 (0.002)	−0.001 (0.022)	−0.000 (0.011)	0.105** (0.050)	−0.017** (0.009)	−0.063** (0.030)	−0.025** (0.012)
Age	−0.005*** (0.001)	0.001*** (0.000)	0.003*** (0.001)	0.001*** (0.000)	−0.000 (0.003)	0.000 (0.000)	0.000 (0.002)	0.000 (0.001)	−0.005*** (0.001)	0.000** (0.000)	0.003*** (0.001)	0.002*** (0.000)	−0.002 (0.002)	0.000 (0.000)	0.001 (0.001)	0.000 (0.000)
Family size	−0.027*** (0.004)	0.003*** (0.001)	0.016*** (0.002)	0.007*** (0.001)	−0.009*** (0.008)	0.001*** (0.001)	0.005*** (0.004)	0.003*** (0.002)	−0.033*** (0.006)	0.002** (0.001)	0.021*** (0.004)	0.010*** (0.002)	−0.029*** (0.007)	0.005*** (0.001)	0.018*** (0.004)	0.007*** (0.002)
Education level	0.016*** (0.003)	−0.002*** (0.000)	−0.010*** (0.002)	−0.004*** (0.001)	0.011** (0.005)	−0.001** (0.001)	−0.007 ** (0.003)	−0.004** (0.002)	0.015*** (0.003)	−0.001** (0.000)	−0.010*** (0.002)	−0.005*** (0.001)	0.003 (0.006)	−0.000 (0.001)	−0.002 (0.003)	−0.001 (0.001)
Reciprocal labor	0.031*** (0.008)	−0.004*** (0.001)	−0.019*** (0.005)	−0.008*** (0.002)	0.037*** (0.035)	−0.003** (0.003)	−0.021*** (0.020)	−0.012*** (0.011)	0.044*** (0.011)	−0.002** (0.001)	−0.028*** (0.007)	−0.014*** (0.004)	0.108*** (0.034)	−0.018** (0.006)	−0.064*** (0.020)	−0.025** (0.009)
*Financial Capital*																
Saving^1^	0.215*** (0.021)	−0.025*** (0.003)	−0.133*** (0.014)	−0.058*** (0.008)	0.261*** (0.034)	−0.025** (0.005)	−0.152*** (0.022)	−0.084*** (0.017)	0.183*** (0.047)	−0.010** (0.004)	−0.116*** (0.030)	−0.058*** (0.017)	0.147*** (0.034)	−0.024*** (0.007)	−0.088*** (0.021)	−0.035*** (0.010)
Borrowings^1^	0.041* (0.022)	−0.005* (0.003)	−0.025* (0.014)	−0.011* (0.006)	0.050 (0.039)	−0.005 (0.004)	−0.029 (0.023)	−0.016 (0.013)	0.010 (0.034)	−0.001 (0.002)	−0.006 (0.022)	−0.003 (0.011)	0.054 (0.036)	−0.009 (0.006)	−0.032 (0.022)	−0.013 (0.009)
*Social Capital*																
Visiting village demonstration	−0.003 (0.023)	0.000 (0.003)	0.002 (0.014)	0.001 (0.006)	0.075** (0.041)	−0.007* (0.004)	−0.044** (0.024)	−0.024** (0.014)	0.019 (0.045)	−0.001 (0.002)	−0.012 (0.028)	−0.006 (0.014)	0.048 (0.040)	−0.008 (0.007)	−0.029 (0.024)	−0.011 (0.010)
Visiting outside village	0.011 (0.030)	−0.001 (0.003)	−0.007 (0.019)	−0.003 (0.008)	0.002 (0.043)	−0.000 (0.004)	−0.001 (0.025)	−0.001 (0.014)	−0.061 (0.052)	0.003 (0.003)	0.038 (0.033)	0.019 (0.017)	0.078 (0.055)	−0.013 (0.009)	−0.047 (0.033)	−0.018 (0.013)
Extension contact	−0.005 (0.005)	0.001 (0.001)	0.003 (0.003)	0.001 (0.001)	−0.004 (0.007)	0.000 (0.001)	0.002 (0.004)	0.001 (0.002)	0.028 (0.022)	−0.001 (0.001)	−0.018 (0.014)	−0.009 (0.007)	−0.006 (0.007)	0.001 (0.001)	0.004 (0.004)	0.001 (0.002)
Information on rainfall^1^	0.037*** (0.021)	−0.004* (0.002)	−0.023** (0.013)	−0.010*** (0.006)	0.112** (0.037)	−0.011** (0.004)	−0.066** (0.022)	−0.036** (0.013)	0.052*** (0.037)	−0.003* (0.002)	−0.033** (0.024)	−0.016** (0.012)	0.081** (0.033)	−0.013** (0.006)	−0.048 ** (0.020)	−0.019 ** (0.008)
Membership in group^1^	0.019*** (0.022)	−0.002** (0.003)	−0.012*** (0.014)	−0.005*** (0.006)	0.068*** (0.035)	−0.006* (0.004)	−0.040*** (0.021)	−0.022*** (0.012)	0.063*** (0.035)	−0.003** (0.002)	−0.040*** (0.022)	−0.020*** (0.011)	0.119*** (0.044)	−0.020* (0.008)	−0.071*** (0.027)	−0.028*** (0.011)
*Physical Capital*																
Distance to nearest Market	0.001 (0.001)	−0.000 (0.000)	−0.000 (0.001)	−0.000 (0.000)	0.002 (0.003)	−0.000 (0.000)	−0.001 (0.002)	−0.000 (0.001)	0.008 (0.006)	−0.000 (0.000)	−0.005 (0.003)	−0.003 (0.002)	0.001 (0.001)	−0.000 (0.000)	−0.001 (0.001)	−0.000 (0.000)
Distance to agriculture office	0.003 (0.002)	−0.000 (0.000)	−0.002 (0.001)	−0.001 (0.001)	0.005 (0.005)	−0.000 (0.001)	−0.003 (0.003)	−0.002 (0.002)	0.009** (0.003)	−0.000** (0.000)	−0.005** (0.002)	−0.003** (0.001)	0.000 (0.003)	−0.000 (0.001)	−0.000 (0.002)	−0.000 (0.001)
Owned radio/TV^1^	0.146*** (0.023)	−0.017*** (0.003)	−0.090 *** (0.014)	−0.039 *** (0.007)	0.156*** (0.037)	−0.015** (0.005)	−0.091*** (0.022)	−0.050*** (0.014)	0.040** (0.048)	−0.002* (0.003)	−0.025** (0.030)	−0.012** (0.015)	0.133*** (0.037)	−0.022*** (0.007)	−0.079*** (0.022)	−0.031*** (0.010)
*Natural Capital*																
Land size (ha)	0.003 (0.003)	−0.000 (0.000)	−0.002 (0.002)	−0.001 (0.001)	0.024 (0.016)	−0.002 (0.002)	−0.014 (0.009)	−0.008 (0.005)	0.001 (0.005)	−0.000 (0.000)	−0.000 (0.003)	−0.000 (0.001)	0.006 (0.005)	−0.001 (0.001)	−0.004 (0.003)	−0.001 (0.001)
Livestock (TLU)	0.016 *** (0.003)	−0.002*** (0.000)	−0.010*** (0.002)	−0.004*** (0.001)	0.030*** (0.007)	−0.003** (0.001)	−0.017*** (0.004)	−0.010*** (0.003)	0.046*** (0.011)	−0.002** (0.001)	−0.029*** (0.007)	−0.014*** (0.004)	0.008** (0.004)	−0.001** (0.001)	−0.005** (0.002)	−0.002** (0.001)
Log likelihood = -2056.4108					−459.13841				−701.43858				−781.42762			
LR Chi^2^ (21)	322.09				178.02				174.24				148.55			
Prob > Chi^2^	0.0000				0.0000				0.0000				0.0000			
Pseudo R^2^	0.0726				0.1624				0.1105				0.0868			
N	1912				516				676				720			

*** at 1% level of significance, ** at 5% level of significance, and * at 10% level of significance, ^1^indicates dummy variables.

#### 4.2.1 Exposure-sensitivity attributes and prevalence of food insecurity

All the variables related to exposure to climate change extremes and damage caused by such extremes (sensitivity) showed a negative relationship with the food-secure category and a positive relationship with the mildly, moderately, and severely food-insecure categories (Table 5). Both aggregate and specific country estimates show that the occurrence of a short or long dry spell during the main production season would decrease the probability that a household would remain in the food-secure category and increase the probability that it would fall into one of the three food-insecure categories. An increase in the frequency of crop loss due to a dry spell would decrease the likelihood of a household being food-secure, and increase the probability that a household would become mildly, moderately, or severely food-insecure. These results were expected, as climate variability has increased the frequency and intensity of extreme events such as erratic rainfall and drought across countries in East Africa (Nicholson, 2017). Consequently, the situation regarding the food security of smallholders and subsistence farm households is worsening in the region (FAO et al., 2018). A study by Tesso et al. (2012) in Ethiopia indicated that annual production losses due to climate variability significantly increase from year to year. Using an IPCC climatic projection model, Schlenker and Lobell, 2010, Zinyengere et al., 2013 noted that agricultural yield losses due to climate variability range from 18% for southern Africa to 22% aggregated across sub-Saharan Africa.

#### 4.2.2 Human /demographic attributes and the prevalence of food insecurity

The human capital-related factors of a household are important determinants of the prevalence status of household food insecurity. Prevalence of household food insecurity may likely vary according to the gender, age and education level of the household head, size of the household, and availability of reciprocal labor^1^ in the household. A male-headed household has a significant positive association with the probability of being in the food-secure category, and a negative association with the probability of being in the mildly, moderately, or severely food-insecure categories in Ethiopia and Tanzania. This result suggests that in Ethiopia and Tanzania, male-headed households provide a better buffer against shortfalls of food access for their household members than their female counterparts. This might be because male-headed households have higher levels of resource endowments than female-headed households in rural areas of Ethiopia and Tanzania. Even if male and female-headed households have equal levels of resource endowments, unobservable characteristics are also responsible for the difference in their food security status. For example, in cases of crop failure due to harsh climatic conditions, cultural and social traditions make it easier for male heads to leave their farms in search of employment elsewhere than for their female counterparts. Due to cultural and social traditions, it is mainly men who acquire agro-climatic information, adopt ideas, and have access to inputs through social groups in East Africa (Kebe et al., 2018). The present result is similar to studies by FAO, 2011, Felker-Kantor and Wood, 2012, Kassie et al., 2014, Abdullah et al., 2019, and Oduniyi and Tekana (2020), that all established that male-headed households were more likely to be food-secure than their female counterparts. Our result contradicts a study by Lutomia et al. (2019) in Kenya, who found a positive relationship between female-headed households and food security. They argued that female household heads provided a more critical buffer against food consumption shortfalls, and that their household members were more likely to be food secure. Other studies also indicate that women traditionally place more emphasis on family and child welfare, often leading to better educational and food-related outcomes (Smith and Haddad, 2000). When women have more decision-making power in the household (e.g., as household head), and a larger share of the household’s total income, the share of the household’s resources that is spent on food increases, leading to more but also higher-quality food (Sraboni et al., 2014). Access and power to control assets are vital pathways to boost income and empower individuals or households to escape from poverty, reduce vulnerability, and adapt and build resilience to accelerating climate change and variability (Ngigi et al., 2016).

Age of the household head in Kenya was negatively associated with the probability of the household being in the food-secure category, while it was positively associated with the probability of being in the mildly, moderately, or severely food-insecure categories. This finding suggests the diminishing contribution of household heads to food security as they age. An increase in age above a certain number of years may reduce the economic contribution of individuals to welfare improvements (Lutomia et al., 2019). Using data collected from 76 low-and middle-income countries, Thome et al. (2019) found that the likelihood of experiencing food insecurity increases with age but decreases with old age (i.e., the slope of age is zero at 45 years old). They argue that those who have reached old age have generally had opportunities that provide for a healthy life, but it may also be because the amount of food one needs declines with age. The present finding is similar to findings by Gebre, 2012, Lutomia et al., 2019, Yahaya et al., 2018; and Oluwatayo and Ojo (2019), who found that the age of the household head was positively related and important in explaining the food-insecure states of households in Ethiopia, Ghana, Kenya, and Nigeria, respectively. The present finding contradicts some studies that argue that as age is a proxy for farming experience, a rural household’s knowledge of food security issues will increase as their heads get older and more experienced in farming and predicting the weather (e.g., Bogale and Shimelis, 2009, Mitiku et al., 2012, Mango et al., 2014).

Family size is found to be highly significant in determining the prevalence of food insecurity in all three countries. This household factor suggests that the probability of being food-secure decreases with an increase in family size, while the probability of being in one of the food insecurity categories increases with an increase in family size. The possible explanation is that in an area where households depend on agricultural land that is less productive due to climate change extremes, an increase in family size results in an increase in demand for food. This demand, however, cannot be matched with the existing diminished levels of household crop production, so ultimately the family becomes food insecure. This finding is similar to those of studies by Bogale and Shimelis, 2009, Ayele et al., 2020. The availability of a person who provides labor for the household significantly determines the prevalence of household food insecurity in all three countries. The result suggests that access to reciprocal labor increases the probability that the household is in the food-secure category, and decreases the probability that it falls in the mildly, moderately, or severely food-insecure categories.

The education level of the household head significantly increases the probability of the household being food secure, and decreases the probability of it being in one of the three food-insecure categories in Ethiopia and Kenya. Education equips individuals with necessary knowledge on how to make a living. Hence, this result indicates the significance of education for households in improving food security, because educated household heads usually practice family planning, thereby limiting their family size and thus increasing their ability to manage the food demands of their households. Moreover, they engage themselves and their family members in various off-farm income-generating activities. Educated farmers are also more likely to adopt drought-resistant agricultural technologies, resulting in higher yields at harvest, and thus enabling their households to become food secure. This result is consistent with findings by Chinnakali et al., 2014, Mutisya et al., 2016, Agidew and Singh, 2018; and Abdullah et al. (2019) in India, Kenya, Ethiopia, and Pakistani, respectively, that all indicate that an increase in education level increases the probability of being food-secure.

#### 4.2.3 Financial and social attributes and prevalence of food insecurity

Rural households’ ability to save money in financial institutions can be an indicator of their improved food security status. These households might have a higher level of resource endowments or surplus production than food-insecure households. If they were to lose their crop because of harsh climatic conditions, they could stabilize their household’s access to food by generating income, withdrawing money, or selling household assets such as livestock. In the present analysis, a household’s ability to save money in financial institutions significantly influences the prevalence of household food insecurity in all three countries. The results suggest that if the current income of the household allows them to save money, they are more likely to be in the food-secure category and less likely to fall in a food-insecure category. On the other hand, a household that is able to borrow money from different sources is also likely to be food-secure and less likely to fall in the mildly, moderately, and severely food-insecure categories. This significant result is consistent with findings by Sharif and Khor (2008); Gupta et al. (2015), and Sam et al. (2018), who noted that the ability to borrow money from various sources such as family members, relatives, and neighbors was considered as a strategy to help households from experiencing insufficiency of food.

Social capital helps households to reduce vulnerability and enhance adaptive capacity and recovery from adverse events (Adger et al., 2009, Bezabih et al., 2013). A social-group-based approach offers alternative sources of livelihood diversification, and acts as a risk-management tool through employing innovative systems to adapt to climate change (Ngigi et al., 2016). It plays a significant role in improving household food security (Mertens et al., 2015, Smith et al., 2017). In the present study, participation in farm demonstrations significantly determined the prevalence of household food insecurity in Ethiopia, while it was not significant in Kenya and Tanzania. The result suggests that participation in village farm demonstrations is positively associated with the probability of being food-secure, and negatively associated with being mildly, moderately, or severely food-insecure in Ethiopia. This result might be connected to the agricultural extension modalities of Ethiopia. Membership of social groups has a positive effect on the probability of being food-secure and a negative effect on being mildly, moderately, or severely food-insecure in all three countries. Using data collected from three agro-ecological zones in Kenya, an analysis by Kebe et al. (2018) indicated that farmers who belonged to social groups were more likely to adapt to climate change, and to change crop variety and type supported by group-based seed acquisition. They became more food-secure than those who were not social-group members. Similar results are found a study by Westengen et al. (2019) in Ethiopia and Tanzania.

Climate variability impacts crop farming systems in different ways, such as damaging crops and causing persistently low yields that could lead to household food insecurity (Sawe et al., 2018). A study by Kinda et al. (2019) suggests that rainfall variability during the cropping season reduces food security in developing countries. Studies by Nhemachena et al., 2010, Charles, 2014, Shumetie and Yismaw, 2017 indicate that rainfall variability and higher average temperatures in Africa negatively affect smallholder farm households’ income that comes from crops and livestock, and as a result, reduces their food security. The present result shows that regular access to information on rainfall and temperature has a positive effect on the probability of households being in the food-secure category and a negative effect on them being in the mildly, moderately, or severely food-insecure categories in all three countries.

#### 4.2.4 Physical and natural attributes and prevalence of food insecurity

Physical and natural capital can provide a buffer for farm households against food insecurity in the face of crop losses due to climate change extremes. Farm households’ ability to cope with or change their food-insecure situation, due to climate change extremes, depends on their access to different assets and infrastructures. We observed how households with a higher number of assets, such as livestock, were able to cope with food shortages (Table 5). The results of the present study indicate that ownership of durable assets such as a radio or television is positively associated with the probability of being food-secure, and negatively associated with being mildly, moderately, or severely food-insecure in all three countries. This might be due to the fact that a household who owns a radio or television regularly receives information on weather forecasts, while households who do not own such items do not.

The results also suggest that increases in the number of livestock (TLU) increase the probability of being food-secure, and decrease the probability of being mildly, moderately, or severely food-insecure in all three countries. This is an indication that ownership of livestock acts as a hedge against food insecurity in the study areas. Livestock, besides its direct contribution to subsistence needs and nutritional requirements, is a vital input into crop production as it provides manure, and serves to accumulate wealth that can be disposed of during times of need, especially when food stocks in the household diminish. This result is consistent with findings by Bogale and Shimelis, 2009, Ayele et al., 2020, who found a negative relationship between TLU and the likelihood of experiencing food insecurity.

## 5 Conclusion and policy implications

Climate variability has increased the frequency and intensity of extreme events such as drought and flooding in East Africa. These events have led to a drastic reduction in agricultural production and household incomes across countries in the region. Reduction in crop production caused by climate change extremes amplifies the existing stress on food insecurity across the region. This natural crisis imposes significant demographic, social, economic, physical, and psychological challenges to households that are primarily dependent on agriculture for their livelihood and food security. However, the degree of exposure and sensitivity to climate change extremes experienced by farm households is not the same, and their capacity to adapt to these extremes varies. Therefore, this study sought to investigate the prevalence of household food insecurity in the face of vulnerability to climate change in East Africa. The study links the FAO concept of food access with the IPCC dimensions (exposure, sensitivity, and adaptive capacity) of climate change vulnerability, using the primary data collected in 2018/19 from three East African countries: Ethiopia, Kenya, and Tanzania. The study went beyond food (in)security as a binary outcome variable, as has been done in many studies, and employed the most sophisticated and innovative food security measure, the HFIAS, to measure food insecurity as an ordinal outcome. An econometric estimation procedure that involves the use of an ordered probit mode was employed to identify the factors affecting the prevalence rates of household food insecurity in three countries.

The results suggest that the occurrence of a short or long dry spell during the main production season would increase the probability that the household fell into one of the food-insecure states in all three countries, and that the loss of crop production due to damage caused by a dry spell would significantly increase the prevalence of food insecurity in the region. The analysis revealed that the adaptive capacity of a household has a significant role in reducing the prevalence of its food-insecurity. The demographic/human, social, financial, physical, and natural attributes of the household play a significant role in reducing the prevalence of food insecurity that is aggravated by climate change extremes in Ethiopia, Kenya, and Tanzania. Strengthening households’ human and financial capital emerged as an influential intervention that would eventually reduce their food-insecure status through improved education of the household head, better family planning, access to reciprocal labor, and increased household savings. An educated farmer or household head is more likely to engage in income-diversification activities, adopt drought-resistant agricultural technologies, achieve a higher level of crop productivity, and become more food secure. Enabling access to information on weather (rainfall and temperature) for agrarian households would be a significant intervention that would help them adapt to climate change extremes and reduce the stress on food security in the region. It would enable farm households to make decisions about planting and harvesting according to the weather conditions. Improved weather and climate information (forecasting) could lead to better agricultural and food security outcomes and benefit farm households in the region. In particular, enabling access to local-level agrometeorological information that realistically relates to the farming environment would be a crucial intervention strategy for coping with climate variability. Delivering seasonal weather forecasts would be an important intervention mechanism to reduce the prevalence of household food insecurity. Building households’ physical and natural capital, such as ownership of a radio, television, and tropical livestock units, would play a significant role in reducing food insecurity in the region.

## Declaration of Competing Interest

The authors declare that they have no known competing financial interests or personal relationships that could have appeared to influence the work reported in this paper.

## Footnotes

1 *Persons who provide labor for other members of the same household (*Hofmeester et al., 2015).
